# The unrelenting tide of osteoarticular infections in children: reflections from Uganda, eastern Africa

**DOI:** 10.5194/jbji-7-183-2022

**Published:** 2022-08-26

**Authors:** Antonio Loro

**Affiliations:** Orthopedic Department, CoRSU Rehabilitation Hospital, P.O. Box 46, Kisubi, Uganda

## Abstract

Forty years ago I made a radical professional choice: to dedicate a few years of practice to the African continent. Not surprisingly, a few years became many. This paper is dedicated to the children who are battling osteoarticular infections and to those who will be struggling with them in future.

## Viewpoint/commentary

When, in December 1982, I disembarked as an aspiring orthopaedic surgeon at Dar es Salaam airport, I thought that I was prepared to cope with the awaiting burden of tropical orthopaedic diseases, where, euphemistically, the word “tropical” stood for something out of the ordinary or different to what I had been taught in medical school.

Before departure I enrolled in courses, had talks with returnees, and researched specific literature. I repeatedly studied the chapters of Maurice King's “Primary Surgery” (King et al., 1990), a pleasant and necessary companion for departing doctors, then and now. Of that book one statement remained engraved in my mind: “Draining pus is still the commonest surgical procedure done in sub-Saharan Africa” (King et al., 1990). A prophetic statement for my career, since managing infections would have required, on average, one-fifth of my surgical time in the following years.

**Figure 1:**
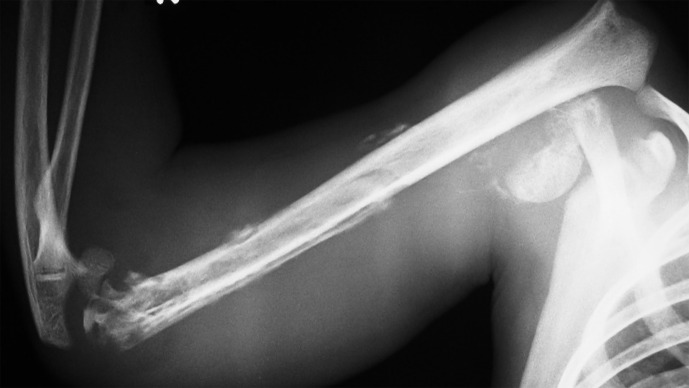
A case of haematogenous osteomyelitis-septic arthritis of the right humerus in an 11-year-old child showing fracture–dislocation of the shoulder, sequestration of the distal half of the diaphysis, and septic arthritis of the elbow joint. The infection was not preceded by any closed or open fracture. A history of blunt trauma prior to the onset was not recorded in this specific case.

When I followed my chief and mentor, Novaretti Giuliano, on the ward rounds in a hospital where orthopaedics was not yet a specialty per se, I began to understand what the word “tropical” meant. Of those poorly ventilated wards hosting all that was septic, I still remember the patients with exposed, necrotic bones, discharging sinuses, and foul-smelling wounds, all of them surgically manageable yet undergoing daily dressing in the meantime. From there, I assume, sprouted my passion for bone and joint infections, with special attention to children with their innocent suffering and resilience. During my first 10 years in Tanzania, my team and I were constantly put to the test with the management of haematogenous infections, due to limited experience and scarce resources (Fig. 1).

Now, in Uganda, where I have been practising for the last 15 years in a well-equipped centre, we are still tested on a daily basis by children presenting with infections that, in the vast majority of cases, are haematogenous in origin. We are still faced with high patient numbers and chronic long-standing cases, not seen any more in the western world, or “peculiar cases”, as a visiting surgeon defined them. The history is repetitive: an onset of a painful swelling, in an otherwise healthy child, followed by fistula formation and pus discharge. While the biological battle between the host and the causative agents unfolds, weeks, months, or even years are lost in traditional treatments, minor surgeries, widespread empirical use of antibiotics, and wound dressings, all measures of dubious and unproven efficacy for infection control. Quite surprisingly, 40 years later, the illustrations of Maurice King et al.'s (1990) book still accurately depict the clinical presentations that we encounter daily in our clinic. Even now, poor accessibility to proper healthcare, both logistically and economically, transforms a curable disease into a permanent disability.

I hardly remember a single case of acute osteomyelitis where timely bone drilling and antibiotics, as we were taught, could eradicate the infection. Most probably these acute cases are still seen at the peripheral level in health facilities where diagnostic and surgical skills may be lacking. The system that should catch and treat the disease at the onset is clearly fragile and inadequate for preventing the evolution towards chronicity (Tamer and Shady, 2021). What we now see in orthopaedic departments, mostly located in major cities, are the devastating sequelae of the natural history of the disease and/or the results of untimely/inadequate treatment. The countryside is where the cases are, and the towns are where the specialists reside. What about the crying demand from neighbouring countries where struggling economies and civil unrest mix with a lack of qualified facilities and human resources?

It still comes to mind how difficult the positioning of our first external fixator was, almost 40 years ago, in a small boy: a torch for light, a manual drill, and four transfixing wires held by wooden bars, eventually reinforced with plaster of Paris. It was a low-cost device, meant for developing countries, whose use was described at that time in a short report in the *Tropical Doctor* journal (Jongen, 1995). What to think of this when I look at my current equipment, mostly donated by colleagues from around the world? We can now rely on equipment that has trickled down from helping hands, and this flow, I hope, will not stop for many years to come. I have witnessed donation of disposable external fixators of every sort, limb reconstruction systems, Taylor's spatial frames, reconditioned drills, and plates/pins of every type and diameter. In some countries, donations (external fixators in primis) have deeply changed the prognosis and outcome of treatment in the last 2 decades.

Here and there, the growing number of orthopaedic and plastic surgeons has done the rest in raising the standards of treatment. In Uganda, we were fewer than 20 specialists in 2006, catering to a population of 28 million; we are now approaching 70. Human resources and up-to-date equipment have made feasible, though a very few select centres, complex reconstructive options, such as bone transport, bone graft, and vascularized flap.

In regions coping with fragile and underfunded health systems, the eradication of infections must still rely on surgery. Surgery, done properly and in a timely fashion, has been, is, and will be the key in the foreseeable future. To what extent the eradication of the infection is achieved by antibiotic therapy is hard to say. Antibiotics are widely prescribed all along the course of the disease, but prescription is mostly done on an empirical basis without microbiological support. In addition, the purchase of the drugs and patient compliance are greatly interwoven with family economic woes. This transforms the accepted role of antibiotic therapy into a debatable point in low-income settings.

There is a need for prospective clinical studies taking into account that the burden is going to remain rooted firmly in the poorest regions of the world. Studies require funding (very often difficult to access), qualified staff, patient compliance with protocols, tracing of the enrolled subjects, and correct data collection and evaluation. In our own experience, however, a lack of or incomplete documentation, unreliable contacts for tracing, and erratic or unaffordable costs of transportation have presented serious obstacles in this specific field.

We all know that the clinical expressions seen here have disappeared from western practices and medical curricula. It is very difficult for a western-trained surgeon to manage conditions that they probably saw and will see only in old books and papers. Do we see on the horizon a new professional figure, a locally trained surgeon, exclusively focused on the unrelenting tide of pediatric osteoarticular infections in the tropics?

Thousands of children continue to suffer from a tragic, preventable disease. Many of them will skip education, and too many will become permanently disabled. Their innocent suffering, their resilience, and their answers to pain are exceptional. What strikes me most is, however, their healing potential. In my old age I am still amazed at witnessing how a skilled surgeon, applying relatively simple procedures, can often overcome devastating bone infections, recreating anatomy and function, even in the presence of poor nutrition and sanitation.

When I look back at our patient cohort, it is clear that they have all come to our attention from areas where the living conditions are poor, sometimes squalid, and always tough. During my stay in Uganda I have barely treated a child coming from middle- or upper-class families. This is just an observation, nothing more, but it speaks volumes about the role played by the economic hardships in this specific field. Clearly, infection and eking out a living with less than USD 3 a day seem to be strictly intertwined in a strong marriage. It appears that rising economies might be the most important factor in preventing and eradicating osteoarticular infections due to their repercussions for nutrition, hygiene, education, and access to health facilities. All the suffering children are not sons and daughters of a capricious, lesser God or hostages of uncontrollable spiritual forces, as is believed in some regions here; more realistically, they are sons and daughters of lesser yet expanding economies.

Is there anything we can do while waiting for an African economic renaissance? The high population growth rate and the soaring number of children will surely strain the existing health facilities. Redistribution of global wealth will take years to happen, and many countries will remain in the developing category for quite a while. Is it too much to hope to have at least one qualified and properly equipped septic unit in each country, acting as a referral point for the most complex cases? This is a task, in some countries a Herculean task indeed, up to the African and global orthopaedic family to achieve.

## Data Availability

No data sets were used in this article.
